# Root Transcriptomic Analysis Revealing the Importance of Energy Metabolism to the Development of Deep Roots in Rice (*Oryza sativa* L.)

**DOI:** 10.3389/fpls.2017.01314

**Published:** 2017-07-26

**Authors:** Qiaojun Lou, Liang Chen, Hanwei Mei, Kai Xu, Haibin Wei, Fangjun Feng, Tiemei Li, Xiaomeng Pang, Caiping Shi, Lijun Luo, Yang Zhong

**Affiliations:** ^1^Department of Ecology and Evolutionary Biology, School of Life Sciences, Fudan University Shanghai, China; ^2^Shanghai Agrobiological Gene Center Shanghai, China; ^3^Shanghai Majorbio Bio-Pharm Technology Co., Ltd. Shanghai, China

**Keywords:** deep rooting, energy metabolism, *Oryza sativa* (rice), QTT (quantitative trait transcripts), RDR (ratio of deep roots), transcriptome, WGCNA (weighted gene co-expression network analysis)

## Abstract

Drought is the most serious abiotic stress limiting rice production, and deep root is the key contributor to drought avoidance. However, the genetic mechanism regulating the development of deep roots is largely unknown. In this study, the transcriptomes of 74 root samples from 37 rice varieties, representing the extreme genotypes of shallow or deep rooting, were surveyed by RNA-seq. The 13,242 differentially expressed genes (DEGs) between deep rooting and shallow rooting varieties (H vs. L) were enriched in the pathway of genetic information processing and metabolism, while the 1,052 DEGs between the deep roots and shallow roots from each of the plants (D vs. S) were significantly enriched in metabolic pathways especially energy metabolism. Ten quantitative trait transcripts (QTTs) were identified and some were involved in energy metabolism. Forty-nine candidate DEGs were confirmed by qRT-PCR and microarray. Through weighted gene co-expression network analysis (WGCNA), we found 18 hub genes. Surprisingly, all these hub genes expressed higher in deep roots than in shallow roots, furthermore half of them functioned in energy metabolism. We also estimated that the ATP production in the deep roots was faster than shallow roots. Our results provided a lot of reliable candidate genes to improve deep rooting, and firstly highlight the importance of energy metabolism to the development of deep roots.

## Introduction

Drought stress is one of the most pressing issues inhibiting global agriculture today (Osakabe et al., 2014). But with the growing of world's population, more food must be produced with less fresh water (Fu et al., 2007; Zhang, 2007). Rice (*Oryza sativa* L.) is the main food for more than half of the world's population. Water deficit may reduce rice production seriously and threaten world food security (Serraj et al., 2009; Luo, 2010; Ahmadi et al., 2014). So, there is an urgent need to understand the underlying physiological and molecular mechanisms of drought resistance to sustain rice production in water-limiting areas (Nguyen et al., 1997; Lanceras et al., 2004; Rabello et al., 2008; Bernier et al., 2009; Serraj et al., 2011). As the main organ to uptake water in soil, root is the key contributor of plants' drought resistance (Kato et al., 2006; Henry et al., 2012). Therefore, recently root has become a hot area of research to improve drought resistance (Coudert et al., 2014).

Deep roots play important role in enhancing plants drought resistance, and it is an important component of roots architecture. According to the research of Uga (Uga et al., 2009; Uga, 2012), the roots distributing at 50–90° with the horizontal are recognized to be deep roots. The deep rooting varieties like upland rice usually have better drought resistance than shallow rooting varieties (Uga et al., 2013a; Lou et al., 2015). Over the course of evolution, the upland rice evolved a lot of adaptive mechanisms to cope with the environmental lack of water, like the feature of deep rooting possessing more and longer roots in deep soil (Kondo et al., 2003; Ding et al., 2011). Increasing deep roots ratio is a promising strategy to improving the drought resistance in rice. In recent years, progress has been made in detecting large effect quantitative trait loci (QTL) conferring the ratio of deep roots (RDR) in rice (Uga et al., 2011, 2012, 2013a,b, 2015; Kitomi et al., 2015; Lou et al., 2015). But, the knowledge about the genetics and molecular control of deep rooting in rice is still relatively limited, for example what genes control the deep rooting in rice and how these genes regulate the deep rooting in rice.

Comparative transcriptomes among specific samples is useful for exploring genes controlling various phenotypes and elucidating genetic mechanisms for plant's adaptation to adverse environments (Gan et al., 2011; Roberts et al., 2011; Yu et al., 2012). The current knowledge of transcriptome of drought resistance in plant mostly related to the comparative studies of different species with diverse genetic background and different ability of drought resistance (Moumeni et al., 2011). In this study, we investigated not only two groups of rice varieties with contrasting roots architecture but also 37 pairs of deep roots and shallow roots from one variety with the common genetic background. Differentially expressed genes (DEGs) were analyzed, and their expression patterns were further confirmed by microarray and qRT-PCR. Through weighted gene co-expression network analysis (WGCNA), we got some hub genes from the co-expressed DEGs, and found that the energy metabolism may play quite important role in the development of deep roots. Furthermore, to compare the difference of ATP synthesis between deep and shallow roots, we measured their oxygen consumption rates and found their rates were significantly different. Nowadays, association mapping has become an important bridge connecting phenotype and genotype to identify important genes controlling traits. So, besides the analysis of DEGs, the association mapping of the quantitative trait transcripts (QTT) with the phenotype was conducted. In our study, 10 QTTs genes associated to deep rooting were identified, and some of them also functioned in energy metabolism.

This study would help us to identify genes and mechanisms involved in the development of roots architecture. More importantly, this study could provide candidate genes to promote molecular breeding and genetic engineering projects for enhancing drought resistance in rice.

## Materials and methods

### Plant materials and growing conditions

**Figure 1:**
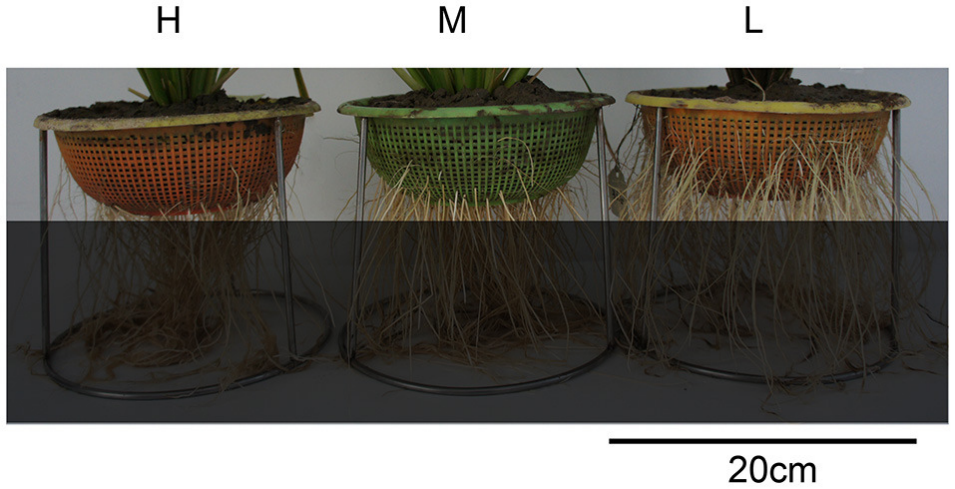
Photos of three types of varieties with different root distribution. H is the deep rooting variety, M is the median rooting variety, and L is the shallow rooting variety.

Through repeated phenotypic validation, 37 rice varieties were screened out from a set of more than 800 varieties (Lou et al., 2015). Fourteen of them are peculiar varieties with extremely deep rooting, fifteen peculiar varieties with extremely shallow rooting, and eight varieties with normally median root distribution (Figure 1, Table S1).

The experiment was conducted in the greenhouse at Shanghai Academy of Agricultural Sciences, Shanghai, China, in 2014 summer. In order to sample roots, the rice seedlings were cultured by hydroponic system using baskets placed in plastic boxes. The composition of nutrient solution was referred to the formula of International Rice Research Institute (IRRI), and the detailed parameters of the basket can be referred to our previous work (Lou et al., 2015). The roots emerging from the bottom and sides were regarded as deep roots (DR) and shallow roots (SR), respectively. For each variety, three baskets were used as replications and two seedlings were planted together in the center of each basket. After growing for 45 days, the tips about 1 cm long of roots were excised and immediately frozen in liquid nitrogen for RNA sampling. About 50 root tips from six plants of each variety were pooled as one sample. Thus, total mRNA of 74 samples (the deep roots sample and shallow roots sample were collected from each of the 37 varieties) was prepared for RNA-seq.

### RNA sequencing

Total RNA was extracted from root tips using Trizol reagent (Invitrogen, USA) and purified using Plant RNA Purification Reagent (Invitrogen, USA). The RNA quality was checked by Bioanalyzer 2100 (Aligent, USA), and the integrity number (RIN) of all these samples's RNA were >9.0.

According to the manufacturer's instructions (Illumina, USA), the sequencing libraries were prepared. Poly-A-containing mRNA was isolated from the total RNA by poly-T oligo-attached magnetic beads, and then fragmented by RNA fragmentation kit. The cDNA was synthesized using random primers through reverse transcription. After the ligation with adaptor, the cDNA were amplified by 15 cycles of PCR, and then 200-bp fragments were isolated using gel electrophoresis. At last, the products were sequenced by an Illumina HiSeq2500 instrument in Majorbio Co. Ltd. (China). The raw data had been submitted to NCBI Sequence Read Archive (SRA) with the Bioproject number PRJNA306542 from SRX1547421 to SRX1547494.

### RNA-seq data analysis

After sequencing, we totally got 3,412,297,344 reads and about 345G bases raw data. After removing Illumina adapter sequences, and trimming low-quality bases, about 306G bases clean data was used in our analysis. Then, we mapped the reads to the Nipponbare MSU7.0 genomic reference (http://rice.plantbiology.msu.edu/index.shtml, Release 7) by the software TopHat v2.0.13 (http://tophat.cbcb.umd.edu/). According to the annotation of MSU7.0, there are 55,963 genes on the rice genome. The screening of DEGs was based on their FPKM (Reads per kilobase of exon model per million mapped reads) values (Mortazavi et al., 2008). The FPKM-value was calculated by the software of cufflinks from the website http://cole-trapnell-lab.github.io/cufflinks/ (Trapnell et al., 2012). The clustering of samples were carried out using the script of clust (method = “average”) in R package. We compared the transcriptomic data in two levels: deep rooting varieties and shallow rooting varieties; and the deep roots and shallow roots from one variety. The comparative analysis of deep rooting varieties vs. shallow rooting varieties was carried out by Wilcox-test using R statistical software (https://www.r-project.org/). The paired comparative analysis of deep roots vs. shallow roots from one variety was conducted by the Cuffdiff software (http://cufflinks.cbcb.umd.edu/). A false discovery rate (FDR) of 0.05 was used for identifying significant DEGs. To inspect the functions of DEGs, the gene KEGG pathway enrichment analysis of the DEGs was performed by the software of KOBAS (http://kobas.cbi.pku.edu.cn/expression.php) and Fisher-test (Benjamini and Hochberg, 1995; Xie et al., 2011). Additionally, the software of Visual Genomics (http://www.vgenomics.cn/) was also used to data analysis and charting.

### qRT-PCR and microarray confirmation

Some DEGs were selected for quantitative confirmation by quantitative real-time PCR (qRT-PCR) analysis with a BIO–RAD CFX96 Thermal Cycler (Bio-Rad, USA) (Livak and Schmittgen, 2001). After reversely transcription by PrimeScript RT reagent Kit (Takara, JP), we obtained the cDNA of the total mRNA. Then, the quantitative PCR reaction used SYBR Premix Ex Taq (Takara, JP). For each gene, the qPCR reactions were performed in biological triplicates.

Twelve 4^*^44K Agilent Rice Oligo Microarray (Agilent Technologies, Inc.) for six rice varieties were performed (Table S2). The samples were planted on June–July in the same place under the same growing condition as that operated in the experiment of RNA-Seq. The microarray experiments were conducted according to Agilent's manuals in SBC (Shanghai Biotechnology Corporation, China).

### QTTs analysis

The association mapping based on the four-omics datasets was called QTX mapping, including quantitative trait SNPs (QTS), QTT, quantitative trait proteins (QTP), and quantitative trait metabolites (QTM) (Zhou et al., 2013). In this study, QTT association mapping was conducted to detect QTT differences on phenotype. The prediction of random effects (q, qq) was obtained using QTXNetwork software based on GPU parallel computation (http://ibi.zju.edu.cn/software/QTXNetwork/). We calculated the QTTs using the transcriptome of deep roots and shallow roots, respectively. After normalized (${\text{log}}_{2}^{\text{X}+1}$), a total of 40,122 mRNA transcripts were used for QTTs mapping. The QTTs of three quantitative traits including SR, DR, and RDR were mapped.

### WGCNA

WGCNA is a system biology method for describing the correlation patterns among genes across a group of special samples. WGCNA can be used for finding modules of highly correlated genes and hub genes with important effect. All the DEGs between shallow roots and deep roots from one variety by paired comparison with *P* < 0.05 were applied to WGCNA. The correlation of co-expressed genes in six different groups was analyzed: including the shallow roots sample (S); the deep roots sample (D); the group of deep rooting rice varieties (H); the group of shallow rooting rice varieties (L); and the group of median rooting rice varieties (M). In this study, the co-expressed genes involved in top 300 most correlated interactions were selected for further analysis (Aoki et al., 2007; Usadel et al., 2009).

### Estimation of ATP synthesis

Oxygen electrodes (Oxytherm, Hansatech) were set up and operated according to the manufacturer's instructions. The reaction solution is 2 ml K_2_HPO_4_ (20 mmolL^−1^) solution at PH = 6.0 and full saturated by air before use. The reaction temperature was set at 25°C, and the speed of magnetic stirrer was set at 100. The reference sequence variety of Nipponbare was used in this experiment, and its fresh deep roots and shallow roots were sampled after 50 days cultivation in green house (25°C, dark 10 h; 35°C, light 14 h). Six pairs of samples of deep roots (D) and shallow roots (S) were replicated, and six intact root tips with 2 cm length were excised for each sample. Then the roots were soaked into reaction solution by injector to remove the surface oxygen and cut into 1 mm long pieces. Put the samples into electrode cuvette, about 5 min later, the oxygen depletion curves were generated to estimate the rates of oxygen consumption. The rate of oxygen consumption could be converted into rates of ATP production, because it was assumed that, in normoxic condition, the ATP:O_2_ ratio was five, based on rates of phosphorylation completed in mitochondria (Chance and Williams, 1956; Gibbs and Greenway, 2003).

## Results

### Gene alignment and sample clustering

After RNA-sequencing, we totally got 295 billion pieces of clean reads, about 43 M reads for each sample (Table S3). On the average, about 92.60% of the reads could be mapped to the reference genomic sequence. And the Q20-values (the base-calling error probabilities = 99%) of the 74 samples are from 91.15 to 94.54%. After filtering, the expression profiles of 40,117 rice genes were used in further analysis.

**Figure 2:**
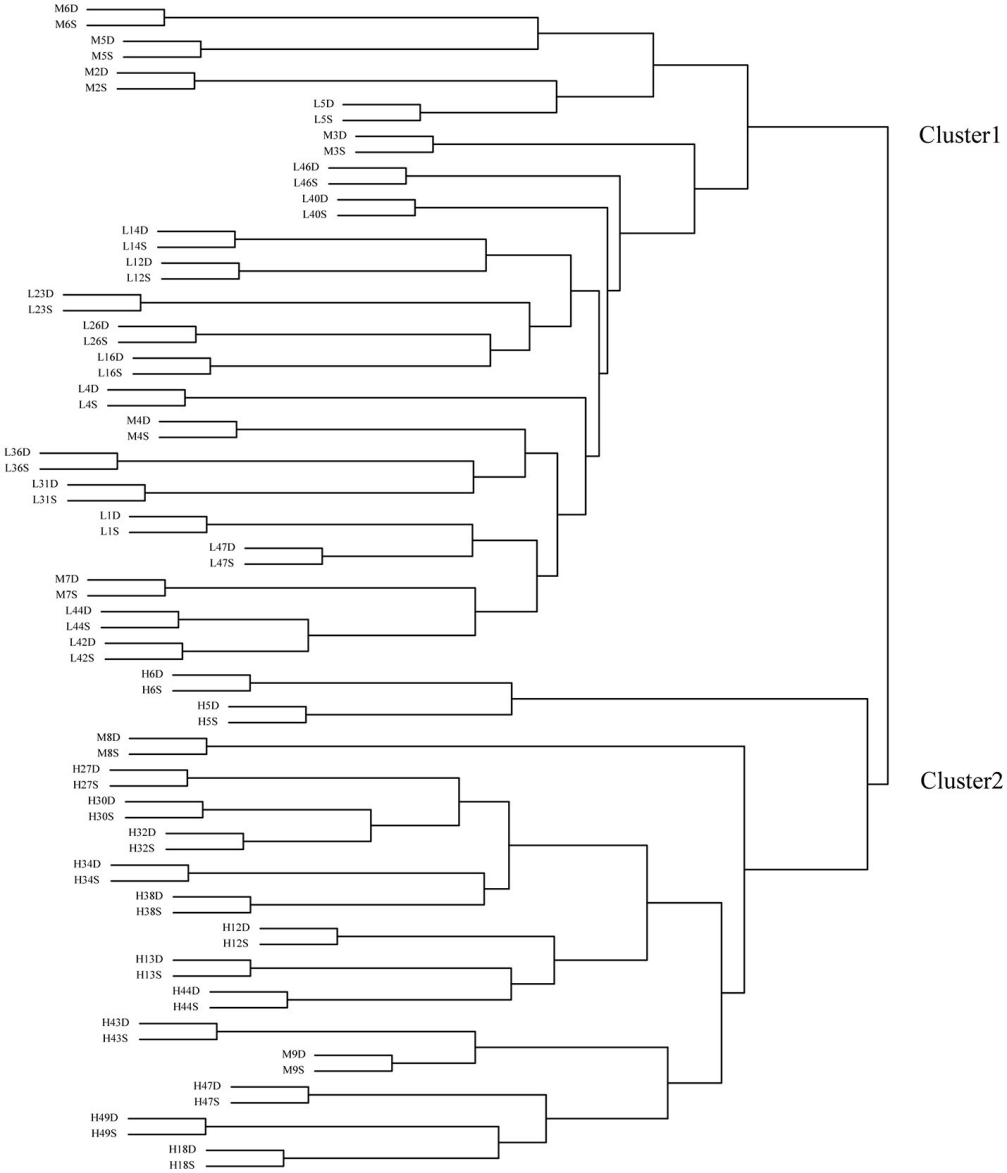
Clustering of the 74 samples by transcriptome data. H is the deep rooting rice varieties, L is the shallow rooting rice varieties, and M is the median rooting rice varieties. S indicates the shallow roots sample (the roots defined by an angle of 50° from the horizontal, centered on the stem of the rice plant), D indicates the deep roots sample (the roots defined by an angle of 40° from the vertical, centered on the stem of the rice plant).

Before clustering, the FPKM-values of the 40,117 genes were normalized by logarithm (${\text{log}}_{2}^{\left(\text{FPKM}+1\right)}$). Then, using these genes' expressing data (FPKM-values), the 74 samples were clustered into two groups (Figure 2). All the deep rooting samples were clustered into one group (Cluster 1), while all the shallow rooting samples were clustered into another group (Cluster 2). Six median rooting varieties (M6, M5, M2, M3, M4, and M7) were located into Cluster 1 and only two median rooting varieties (M8, M9) were clustered into Cluster 2. The separate clustering of shallow and deep rooting varieties exhibited large difference at gene expression level between rice varieties with different root architectures.

Each pair of shallow roots and deep roots samples from the same variety was always stayed together in the clustering tree. This result was in full accordance with the clustering result by microarray (Figure S1). It indicated the genes' expression differences between roots with different spatial locations from the same variety were much smaller than the differences among roots from different varieties.

### DEGs between the deep and shallow rooting varieties

**Figure 3:**
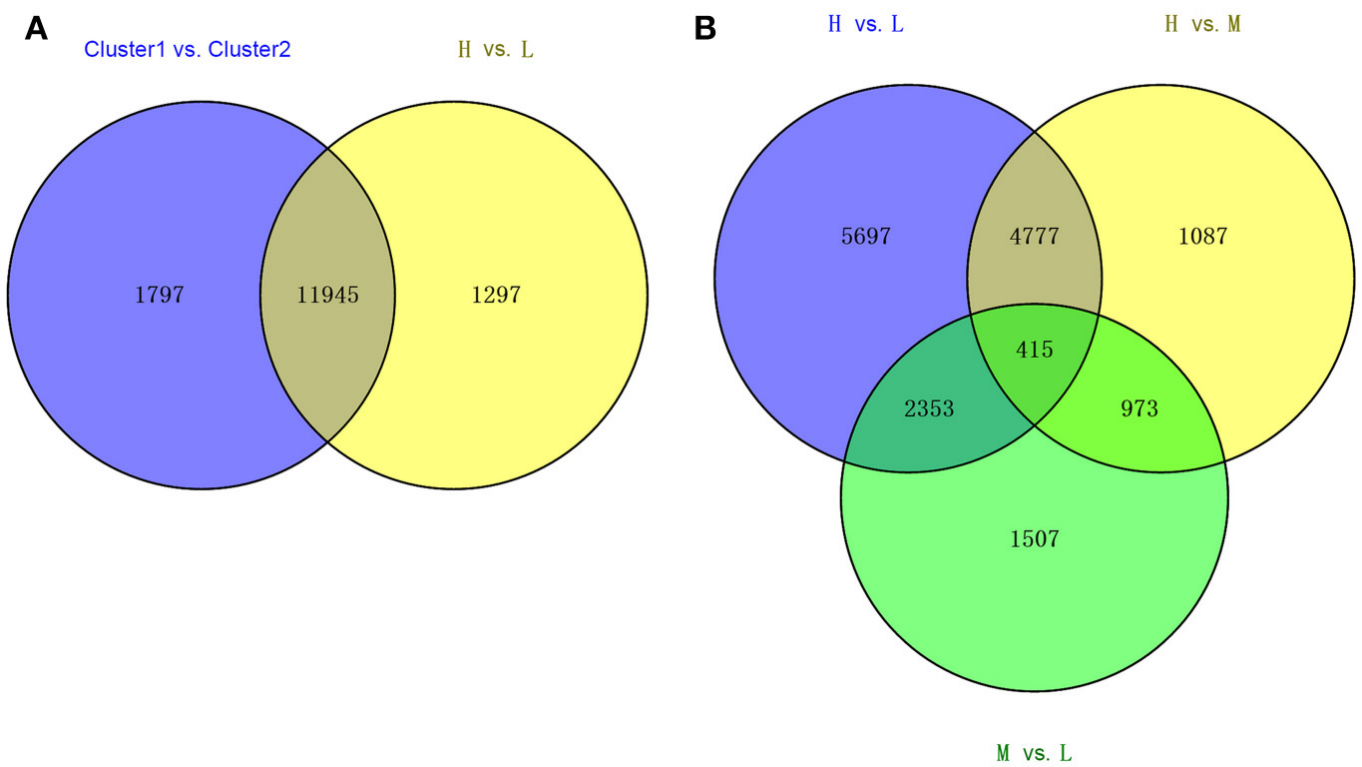
The Venn diagram of the different collections of DEGs. **(A)** The overlaps of DEGS from Cluster1 vs. Cluster2 and the DEGs from H vs. L. **(B)** The overlaps of DEGs from three groups' comparison: H vs. L, H vs. M, and M vs. L. Cluster1 and Cluster2 was classified by clustering analysis referred to the Figure 2. H is the deep rooting rice varieties, L is the shallow rooting rice varieties, and M is the median rooting rice varieties.

Using the statistic method of Wilcox-test, we compared the transcriptomes of the deep rooting varieties to shallow rooting varieties with the criterion at FDR < 0.05. A total of 13,742 DEGs were detected between above-mentioned Cluster 1 vs. Cluster 2, while 13,242 DEGs were found between the deep rooting varieties compared with shallow rooting varieties (H vs. L) (Figure 3A). And there were 11,945 common DEGs that occupied about 90% of the total DEGs of H vs. L, so the comprehensive comparison between H and L could represent the comparison in all varieties. Furthermore, the DEGs of H vs. L, H vs. M (deep rooting varieties compared with median rooting varieties), and L vs. M (shallow rooting varieties compared with median rooting varieties) were pair-wise compared (Figure 3B). The DEGs of H vs. L was more than the twice of L vs. M. There were 5,697 DEGs exclusively detected in H vs. L, while only 1,087 and 1,507 DEGs exclusively detected in H vs. M and L vs. M. The difference of gene expression pattern between deep rooting varieties and shallow rooting varieties was much greater than their difference with median rooting varieties.

Furthermore, we screened the DEGs using some criterions (Table 1). Firstly, the fold changes (average FPKM-values of H/L) were applied to screen candidate genes, and obtained 4,185 DEGs with fold changes >2: containing 1,634 DEGs with H/L < 0.5 and 2,551 DEGs with H/L > 2. It indicated more than half of the DEGs expressed higher in deep rooting varieties than shallow rooting varieties. Secondly, when considered the reliability of the data, the DEGS with high expression (average FPKM-values > 0.5) were selected for further analysis. There were 1,789 DEGs with average FPKM-values > 0.5 at least in one group (H and L): containing 1,403 DEGs with average FPKM-values > 0.5 in one of H or L group and 386 DEGs with average FPKM-values > 0.5 both in H and L group. In the end, these 1,789 genes most likely associated to roots' distribution were selected for enrichment analysis.

**Table 1:** The list of differentially expressed genes (DEGs) between deep rooting and shallow rooting varieties under different criteria.

			**Number of DEGs**
FDR < 0.05			13,242
	Fold changes > 2		4,185
	H/L > 2		2,551
	H/L < 0.5		1,634
		Average FPKM > 0.5	1,789
		H or L > 0.5	1,403
		Both H and L >0.5	386

Based KEGG analysis (Kyoto Encyclopedia of Genes and Genomes, http://www.kegg.jp/), 10 pathways were significantly enriched using the 1,789 DEGs (Table 2). The first four all belong to the metabolism class involved in genetic information processing like DNA replication and mismatch repair. Another four pathways related to metabolism were significantly enriched too. Therefore, the difference in the genetic information processing and metabolism could distinct the deep rooting and shallow rooting varieties, and they contributed greatly to the differentiation of roots distribution in rice.

**Table 2:** KEGG pathway enrichment analysis of 1,789 genes differentially expressed between deep rooting and shallow rooting rice varieties.

**KEGG pathway**	**ID**	***P*-values**	**Classification**
DNA replication	ko03030	0.000657	Genetic information processing
Mismatch repair	ko03430	0.000669	Genetic information processing
Nucleotide excision repair	ko03420	0.002917	Genetic information processing
Homologous recombination	ko03440	0.0041	Genetic information processing
Metabolism of xenobiotics by cytochrome P450	ko00980	0.007219	Metabolism
Drug metabolism—cytochrome P450	ko00982	0.007219	Metabolism
Plant-pathogen interaction	ko04626	0.021512	Organismal systems
MAPK signaling pathway	ko04010	0.028472	Environmental information processing
Phenylpropanoid biosynthesis	ko00940	0.045928	Metabolism
Phenylalanine, tyrosine and tryptophan biosynthesis	ko00400	0.049828	Metabolism

*The DEGs used here meet three criteria: FDR < 0.05, fold changes > 2 and average FPKM > 0.5 as this table shows*.

### DEGs between the deep and shallow roots from the same variety

In three group of rice varieties with deep, shallow and median rooting, totally 1,052 DEGs were detected with FDR < 0.05 between deep and shallow root samples (D vs. S) (Table S4). This number was far smaller than the DEG number of H vs. L. Only 599, 488, and 299 DEGs of D vs. S were identified in deep, shallow and median rooting varieties groups, respectively. Most of the DEGs have the same expression pattern in different varieties in the same group. In the deep rooting and median rooting group, more than half of the DEGs were up regulated especially in the median rooting varieties, while that was just the opposite in the shallow rooting varieties. These DEGs were enriched significantly in 18 KEGG pathways with *P* < 0.05, and 17 of them belong to metabolism pathways especially the energy metabolism (Table S5).

To narrow the candidate genes, the DEGs could be repeatedly detected in different rice varieties were chosen for further analysis. Table 3 demonstrated the genes that could be repeatedly detected in more than three pairs of roots samples. More than half of the DEGs in Table 3 were down regulated in H and L group, suggesting the repeatedly detected DEGs expressed higher in deep roots than in shallow roots in H and L rice groups. But in the median group (M), it was completely opposite because all the DEGs were expressed higher in shallow roots than in deep roots except LOC_Os11g24140. Nine genes in Table 3 could be detected both in at least four deep and four shallow rooting varieties, and three genes can be detected both in more than three median and four shallow rooting varieties. The gene of LOC_Os02g37190 could be detected in all three groups. In Minghui 63 rice variety, LOC_Os02g37190 expressed higher in roots than all other tissues of plant (Wang et al., 2010). Four genes in Table 3 (LOC_Os02g48710, LOC_Os05g48890, LOC_Os04g56100, and LOC_Os09g00999) appeared only in a single group. All of them were detected only in the shallow rooting varieties group, so these genes might exclusively function in the shallow rooting varieties. Furthermore, it was interesting to find almost all the genes expressed the same trend in different varieties within a group, no matter the deep rooting varieties or the shallow rooting ones. For example, LOC_Os01g57958 gene was down regulated both in 10 H varieties and in five L varieties. It suggested these genes have no variety-specificity, and they belong to organ-specificity genes.

**Table 3:** The number of differentially expressed genes (shallow roots vs. deep roots) that could be repeatedly detected in more than three varieties.

**Gene ID**	**Ups^H^**	**Downs^H^**	**Ups^L^**	**Downs^L^**	**Ups^M^**	**Downs^M^**
LOC_Os01g07850			4	0	4	0
LOC_Os01g22670					3	0
LOC_Os01g57958	0	10	0	5		
LOC_Os01g57968	0	8	0	6		
LOC_Os01g71340	3	3	1	3		
LOC_Os02g37190	4	0	4	0	3	0
LOC_Os02g37250					3	0
LOC_Os02g48710			4	0		
LOC_Os03g05640			0	4		
LOC_Os03g31750			0	6		
LOC_Os04g16722	0	10	0	7		
LOC_Os04g56100			7	0		
LOC_Os05g15880	0	5	1	3		
LOC_Os05g48200			0	7		
LOC_Os05g48890			5	0		
LOC_Os06g31960			4	0		
LOC_Os07g25050					3	0
LOC_Os07g25060					3	0
LOC_Os09g00999			0	8		
LOC_Os09g01000	0	7	0	7		
LOC_Os09g24370					4	0
LOC_Os10g21346			0	5		
LOC_Os10g21372	0	7	0	7		
LOC_Os11g24140	3	5	0	6	1	3
LOC_Osm1g00500	0	6				
LOC_Osp1g00770	0	4				
LOC_Osp1g01040			0	4		

*Ups were the up regulated genes that expressed higher in shallow roots than in deep roots; Downs were just the opposite. Superscript H is the group of deep rooting rice varieties, superscript L is the group of shallow rooting rice varieties and superscript M is the group of median rooting rice varieties*.

### DEGs related to root development

According to the review of Jung and Mccouch (2013), totally about 1,380 candidate genes may control the architecture development of roots in rice. In this study, 1,052 DEGs were found between the shallow roots and the deep roots from the same variety as above analysis. And 75 of the DEGs were overlapped with the 1,380 genes that had been reported to be responsible for the roots architecture (Tables S6, S7). Through χ^2^-test, we could found these DEGs were significantly enriched in the collection of the genes controlling the architecture of roots in rice, and the *P*-value (asymptotic significance two-sided) of Pearson Chi-Square-test was little to 8.04E-22.

To further study the 75 overlapped genes that related to roots architecture, we quantified the expression of these genes in two rice varieties: H32 and L16 using microarray and qRT-PCR. Forty-nine genes of the 75 overlap genes were detectable by at least two methods. Table 4 indicated these 49 genes' expression ratio in shallow roots to deep roots (S/D) measured by RNA sequencing, qRT-PCR, and microarray. Almost all genes had the same expression trend among the three methods, and only a few genes' expressions measured by microarray were inconsistent. The correlation coefficients of these DEGs expression profiles obtained by different methods were all much larger than the significant threshold of 0.28 (Table S8). It suggested the reproducibility among these three methods of transcriptomic surveying was excellent, and the expression patterns of the DEGs were stable even using the samples cultured in different time. Between the deep rooting variety (H32) and the shallow rooting variety (L16), some genes had different expression trend, for example LOC_Os10g39130 expressed significantly higher in the shallow roots of H32 than that in L16. The average values of all the genes' expression ratios of s/d were near 1, because the expression directions of the 49 DEGs were different and neutralized by each others. In the results of RNA-Seq and qRT-PCR, the average ratios of s/d in L16 (1.26 vs. 1.03) were a little larger than that in H32 (1.01 vs. 0.90), suggesting these genes might express higher in shallow roots of shallow rooting variety than that in deep rooting variety.

**Table 4:** Verification of 49 candidate genes relating to rice roots architecture by real time PCR quantification and RNA microarray.

	**RNA-seq**		**qRT-PCR**		**Chip**	
	**H32s/d**	**L16s/d**	**H32s/d**	**L16s/d**	**H32s/d**	**L16s/d**
LOC_Os01g04800	1.14	0.79	1.37	0.76	1.53	1.40
LOC_Os01g10040	1.28	1.18	1.84	1.55	1.70	1.06
LOC_Os01g42380	1.06	0.54	1.09	0.64	1.18	0.31
LOC_Os01g50820	0.66	0.87			0.71	0.44
LOC_Os01g58240	0.84	0.59	0.65	0.42	0.94	0.97
LOC_Os01g58290	1.23	2.17	1.20	1.89	2.13	0.75
LOC_Os01g63980	0.36	0.24	0.44	0.18	0.52	0.44
LOC_Os01g72140	0.62	0.74	0.31	0.46	0.39	0.56
LOC_Os01g72550			1.16	1.05	1.02	1.13
LOC_Os02g02190	0.31	3.47	0.25	1.02	1.76	0.55
LOC_Os02g11760	1.20	0.87	1.14	0.83	1.64	0.62
LOC_Os02g17900	0.51	0.12	0.25	0.14	1.74	0.28
LOC_Os02g20360	0.74	0.68	0.76	0.54	0.90	0.79
LOC_Os02g32590	0.69	0.57	0.55	0.67	0.35	0.38
LOC_Os02g40710	0.70	0.46			0.88	0.44
LOC_Os02g49440	1.03	1.45			0.79	1.01
LOC_Os03g08754	0.99	2.04	0.63	1.59	0.87	0.95
LOC_Os03g09880	0.49	0.59	0.18	0.23	0.77	0.72
LOC_Os03g49260	1.20	0.92	1.23	0.79	1.61	1.33
LOC_Os03g50960	0.49	0.41			0.66	0.60
LOC_Os03g52860	1.08	2.16	1.73	2.04	2.07	3.59
LOC_Os03g60570	0.78	0.57			0.33	0.34
LOC_Os04g08350	2.23	1.06	1.24	1.46	1.00	1.49
LOC_Os04g33900	1.60	3.29			0.67	0.48
LOC_Os04g44060	0.62	0.87	0.42	0.94	0.83	1.14
LOC_Os04g46810	1.74	2.31	1.56	1.17	3.33	4.95
LOC_Os04g46820	1.92	2.58	1.87	1.04	3.41	4.83
LOC_Os04g49150	0.00	2.59	0.09	2.73	1.03	2.32
LOC_Os04g51460	0.88	0.96	0.75	0.73	2.33	0.52
LOC_Os05g02070	0.96	0.86	0.58	0.67	0.54	0.52
LOC_Os05g45410	0.84	0.68	0.51	0.67	0.71	0.76
LOC_Os05g48890	1.72	2.16	1.89	1.75	1.40	1.93
LOC_Os06g06350	0.95	0.95	0.68	0.51	0.71	1.33
LOC_Os06g36850	0.00	1.45	0.14	1.60	0.54	1.34
LOC_Os06g48200	0.96	1.97	0.84	1.69	0.79	1.05
LOC_Os07g39320	1.68	0.79			1.65	0.90
LOC_Os08g02070	0.78	0.84	0.66	0.48	0.43	0.56
LOC_Os08g24790	0.97	1.32			0.47	0.97
LOC_Os08g37210	1.11	0.52	0.93	0.90	1.93	1.02
LOC_Os08g37250	0.63	0.91	0.47	0.74	0.87	0.64
LOC_Os08g41720	1.24	0.68	1.67	0.87	1.93	0.25
LOC_Os08g44270	0.78	1.36	0.56	0.86	0.74	0.69
LOC_Os10g07510	1.64	0.84			1.67	1.02
LOC_Os10g38740	0.83	1.14			1.09	5.28
LOC_Os10g39130	3.40	0.84	1.82	0.48	1.46	0.73
LOC_Os10g40520	1.38	1.16			1.58	1.06
LOC_Os11g16970	0.85	1.15			0.89	1.81
LOC_Os11g18366	0.78	2.94	0.70	1.88	0.92	1.12
LOC_Os11g45280	1.58	2.95	1.27	1.57	2.35	1.96
Average	1.03	1.26	0.90	1.01	1.22	1.21

*s/d is the ratio of gene's expression in shallow roots contrast to deep roots*.

### Quantitative real-time PCR validation of candidate genes

**Figure 4:**
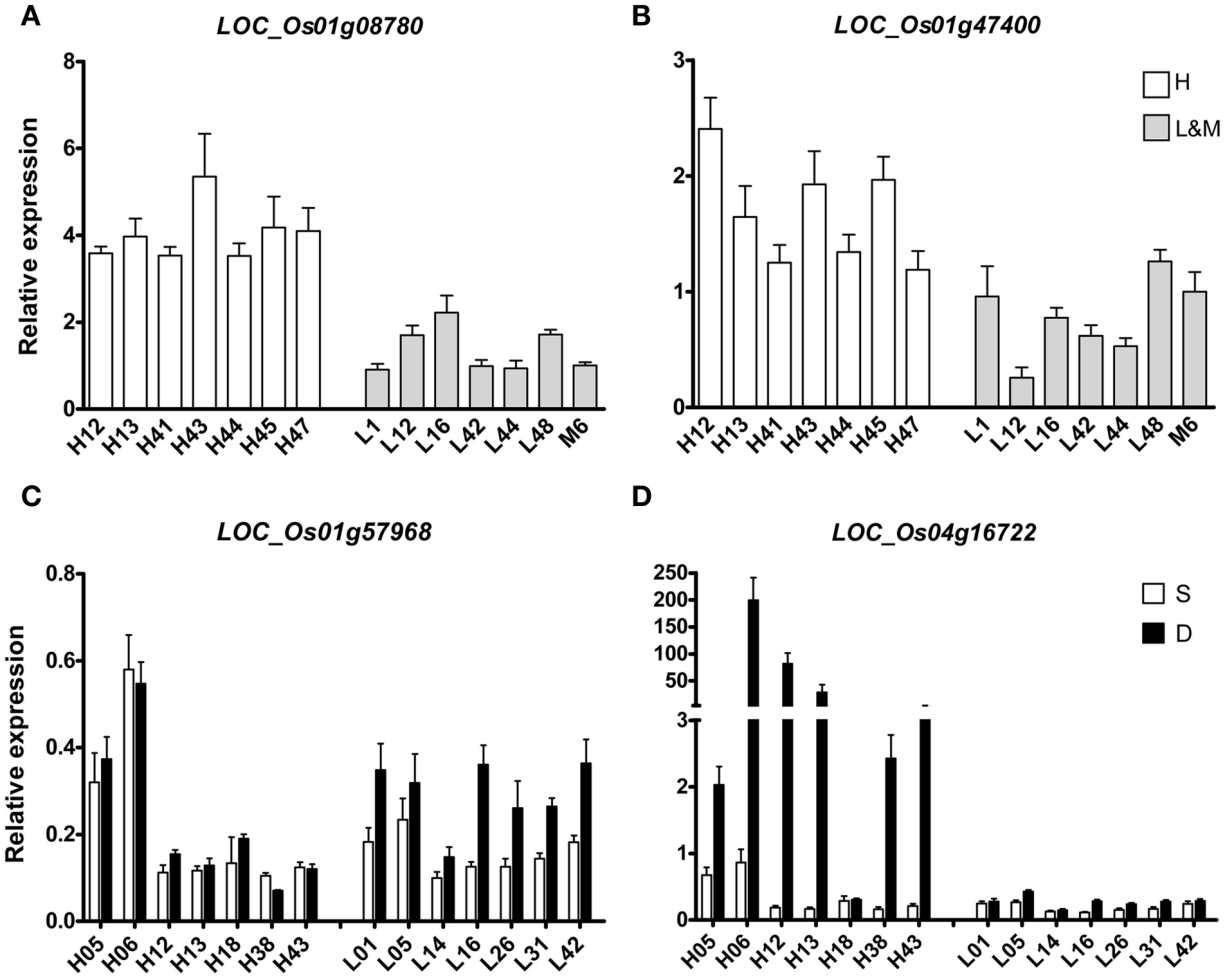
qRT-PCR of four candidate DEGs in the roots of 14 rice varieties. **(A,B)** indicated two DEGs that differentially expressed between deep rooting and shallow rooting varieties; **(C,D)** indicated two DEGs that differentially expressed between deep roots and shallow roots from one variety.

To further verify and study the function of some putative genes, four DEGs were chosen for qRT-PCR-test. Two DEGs (LOC_Os01g47400 and LOC_Os01g08780) of H vs. L that were located on the known QTL intervals controlling RDR had been reported in our previous study (Lou et al., 2015) were carried out qRT-PCR-test in 14 rice varieties that were randomly chosen from all the rice varieties (Figures 4A,B). LOC_Os01g47400 is an Osman01-endo-beta-mannanase, belongs to glycosyl hydrolase superfamily. LOC_Os01g08780 contains the phosphatase family domain, behaving as an inositol polyphosphate 5-phosphatase I. LOC_Os01g47400 and LOC_Os01g08780 both expressed differently in H and L groups. Their mRNA levels were significantly higher in H group than in L group varieties. So the activity of these two enzymes in deep rooting varieties may be higher than that in shallow rooting varieties.

Two other DEGs (LOC_Os01g57968 and LOC_Os04g16722) of D vs. S from Table 3 were selected for qRT-PCR-test in 28 RNA samples including 14 deep roots samples and 14 shallow roots samples (Figures 4C,D). The results of qRT-PCR coincided with the results of transcriptomic sequencing. In some rice varieties, those two genes both expressed differently in shallow roots and deep roots. The relative mRNA level of LOC_Os01g57968 in the deep roots was higher than its expression in the shallow roots, and their expression differences were especially dramatical in the shallow rooting group (L). But, for the gene of LOC_Os04g16722 with ycf68 domain, its transcriptomic difference was more significant in the deep rooting varieties group (H) than in the shallow rooting varieties group (L). Those candidate genes were in the process of genetic function verification by transgenic test.

### QTT mapping

The QTT module in QTXNetwork was applied to screen significant transcripts and to predict their effects on phenotype. There were a total of 10 QTTs that were detected to control the vertical distribution of roots in rice (Table 5). Four of them were overlapped with the DEGs between deep rooting and shallow rooting varieties. Seven QTTs were identified in the deep roots, and three QTTs were found in the shallow roots. There was no overlap between the QTTs of deep roots and shallow roots. Most of the QTTs detected in this study were single gene locus, and only one of them had qq interactions. The qq interaction of LOC_OS01G15860 × LOC_OS05G49980 might affect the DR (the number of deep roots) of rice with the *h*^2^ of 42%.

**Table 5:** Estimated heritability of QTTs for three traits of Root.

**Transcriptome**	**Trait**	**Locus**	**Q/QQ**	***P*-value**	***h*^2^**
Deep roots	DR	LOC_Os01g15860 × LOC_Os05g49980	122.22	1.60E-14	0.42
		LOC_Os01g02280	141.04	2.19E-18	0.56
	SR	LOC_Os01g65150	235.13	9.52E-05	0.29
		LOC_Os10g28400	353.52	1.51E-05	0.65
	RDR	LOC_Os01g42430	12.10	2.76E-03	0.23
		LOC_Os03g44840	15.69	1.66E-03	0.39
		LOC_Os08gG06810	−10.19	5.85E-04	0.17
*Shallow roots*	*SR*	*LOC_Os01g42140*	*543.71*	*3.89E-08*	*0.95*
	*RDR*	*LOC_Os03g08230*	*−33.02*	*8.20E-10*	*0.61*
		*LOC_Os09g10860*	*24.19*	*1.26E-05*	*0.33*

*Q/QQ: Additive effect on the phenotype when the expression of the gene increasing two units. Two sets of transcriptome data were calculated in this study, the first half of the table showed the results of QTTs mapping using the deep roots transcriptomes, the second half of the table (italic effect) showed the results of QTTs mapping using the shallow roots transcriptomes*.

RDR was the RDR, so it was decided by the number of deep roots and shallow roots. Three genes (LOC_OS01g42430, LOC_OS03g44840, and LOC_OS08g06810) in deep roots and two genes (LOC_OS03g08230 and LOC_OS09g10860) in shallow roots were identified to influence the RDR. Four genes of the QTTs (LOC_Os01g42430, LOC_Os01g65150, LOC_Os05g49980, and LOC_Os10g28400) were also found to be differentially expressed between deep rooting and shallow rooting varieties (Table S9), and two of them (LOC_Os01g42430 and LOC_Os01g65150) were annotated to be related to energy metabolism: LOC_Os01g42430 contributes to energy production while LOC_Os01g65150 relates to energy consumption.

### Co-expressed genes network

WGCNA could find the network of associated genes with certain functions and identify new genes (Brown et al., 2005). We analyzed the co-expression network of DEGs in 6 different groups of samples. Totally 9820 DEGs of D vs. S with *P* < 0.05 were used in WGCNA. After analysis, the top 300 interactions of correlated DEGs were chosen for further analysis (Table S10). The average weights of the top 300 co-expressed interactions in these six groups ranged from 0.44 to 0.65. In shallow rooting materials, the correlations among the DEGs were much closer than any other groups.

**Figure 5:**
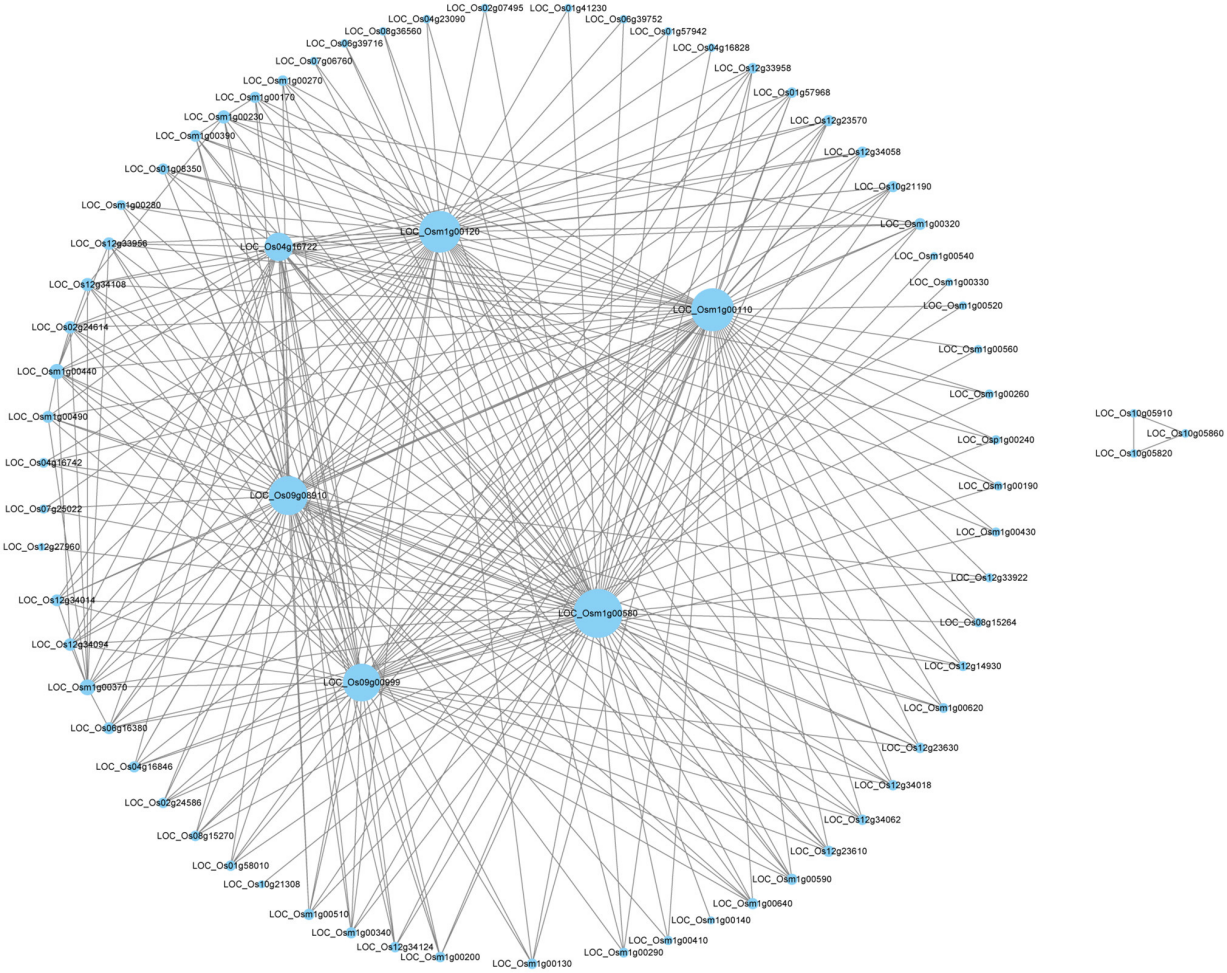
The network of the top 300 co-expressed genes pairs from WGCNA. The WGCNA (weighted gene co-expression network analysis) used all DEGS of deep roots vs. shallow roots with *P* < 0.05 in whole samples.

Through WGCNA analysis, one big module having comprehensive interaction was found, and almost all the genes were clustered into this big module (Figure 5). Inside of the module, there were some hub genes having many co-expressed genes. Table 6 indicated the information of the hub genes with ≥7 significantly co-expressed genes. From this table, two obvious phenomena could be found: (1) half of the hub genes are from the mitochondria genome; (2) more than half of the hub genes relate to energy metabolism, especially relate to ATP biosynthesis. Furthermore, except LOC_Os12g34108, all the 17 hub genes in Table 6 expressed significantly higher in deep roots than in shallow roots. So we can infer that the deep roots have stronger energy metabolism compared with the shallow roots. Besides the genes related to energy metabolism, the other hub genes belong to two other types: five hub genes of them act on transcription and translation, and three hub genes are uncharacterized proteins.

**Table 6:** The annotation of the hub genes with ≥7 co-expressed genes identified by WGCNA.

**Node**	**Num**	***P*-value**	**Fold change**	**Gene annotation**	**GO term**
**LOC_Osm1g00580**	63	5.80E-03	6.14	ATP synthase F0 subunit 1	**ATP biosynthetic process**
**LOC_Osm1g00110**	54	4.40E-03	10.73	Cytochrome c oxidase subunit 3	**Respiratory electron transport chain**
**LOC_Osm1g00120**	52	7.90E-03	9.79	Hypothetical protein	**ATP synthesis coupled proton transport**
LOC_Os09g08910	48	3.42E-03	4.63	ATP synthase subunit alpha	**Response to stress**
LOC_Os09g00999	46	2.76E-03	4.62	Putative uncharacterized protein P0459B01.16	Unannotated
LOC_Os04g16722	32	2.17E-03	4.85	Uncharacterized protein ycf68	Unannotated
**LOC_Osm1g00370**	13	2.48E-03	6.77	ATP synthase F0 subunit 6	**ATP synthesis coupled proton transport**
**LOC_Osm1g00440**	12	1.80E-03	6.37	Ribosomal protein L2	*Translation*
LOC_Os12g34108	9	8.80E-02	1.39	ATP synthase protein 9	**Transporter activity**
**LOC_Osm1g00230**	9	8.73E-03	7.19	NADH dehydrogenase subunit 7	**Oxidation-reduction process**
LOC_Os02g24614	8	1.48E-02	3.53	DNA-directed RNA polymerase subunit beta	*Transferase activity*
LOC_Os12g33956	8	1.28E-02	3.19	Maturase	*Transferase activity*
LOC_Os12g34094	8	8.04E-03	4.66	NADH-ubiquinone oxidoreductase chain 4	**Generation of precursor metabolites and energy**
LOC_Os06g16380	7	7.97E-03	8.51	Expressed protein	Unannotated
LOC_Os12g34014	7	7.52E-03	10.62	NADH-ubiquinone oxidoreductase chain 6	**Generation of precursor metabolites and energy**
**LOC_Osm1g00320**	7	2.60E-02	5.29	NADH dehydrogenase subunit 4	**Oxidation-reduction process**
**LOC_Osm1g00390**	7	2.74E-02	10.18	Ribosomal protein S13	*Translation*
**LOC_Osm1g00490**	7	1.04E-02	3.03	Maturase-related protein	*mRNA processing*

*The boldface in the “Node” column highlighted the genes located on the mitochondria genome. Num, is the number of co-expressed genes with the node gene in the first column; P-value, is the significance of the paired t-test of the gene expression between 37 pairs of deep roots and shallow roots in the same variety; Fold change, the average fold changes of the 37 pairs of gene expressions between the deep roots and shallow roots from the same variety. The boldface in the “GO Term” column highlighted the genes related to energy metabolism, and the italic highlighted the genes related to transcription and translation*.

### Estimation of ATP synthesis

To estimate the rates of ATP synthesis, we measured the rate of oxygen consumption in roots for there were fixed ratio between the oxygen consumption and ATP synthesis in respiration reaction (Greenway and Gibbs, 2003; Edwards et al., 2012).

**Figure 6:**
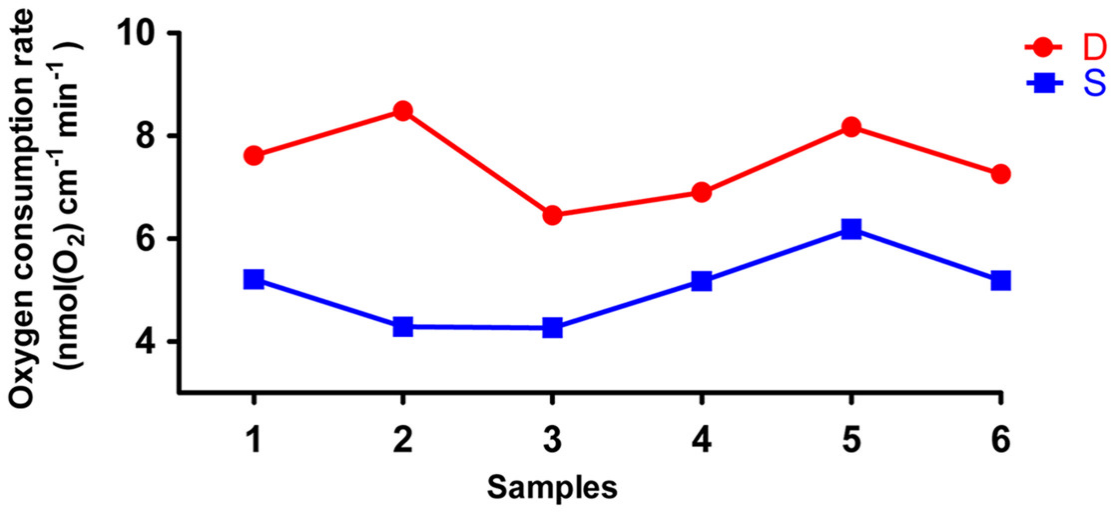
The oxygen consumption rates of six pairs of deep roots and shallow roots. D is deep roots and S is shallow roots. The 1–6 at X-axis is the six replicates of the samples.

The rates of O_2_ consumption between the deep roots and the shallow roots from the same plants were significantly different, and the deep roots used more oxygen than shallow roots in all six pairs of samples (Figure 6). The average rate of oxygen consumption of the deep roots was approximately 7.5 nmol(O_2_) cm^−1^ min^−1^, and the average rate of oxygen consumption of the shallow roots was about 5.1 nmol(O_2_) cm^−1^ min^−1^. The *P*-value of paired student test of the rates of oxygen consumption between deep and shallow roots is 0.001. So, we could conclude the rates of ATP production between deep roots and shallow roots were different, and the ATP production in the deep roots was faster than shallow roots.

## Discussion

Fresh water shortage is a global challenge for all countries. In China, agriculture used about 70% of the total water consumption of the country, 70% of which was used for rice production alone (Zhang, 2007). As the main water consumers, rice is more susceptible to drought stress than other crops. Enhancing crops' drought resistance through genetic improvement has proved to be practicable to obtain stable and adequate crop production in drought-prone areas (Wang et al., 2005; Gowda et al., 2011). The experiments of Uga et al. (2013a) suggested that increasing the RDR would contribute obviously to drought avoidance in rice. Some QTLs controlling RDR in rice have been reported (Uga et al., 2011, 2013b, 2015; Kitomi et al., 2015; Lou et al., 2015). Our research firstly profiled the transcriptions of root samples with diverse RDR and different spatial distribution underground. The DEGs of this study come from two levels of transcriptomic comparison: first comparison among the three groups of varieties with different roots distribution, second comparison between the paired roots samples (D vs. S) in the same variety. Compared with previous transcriptomic experiments, this experiment should be more accurate and meaningful, firstly because amounts of samples with different RDR were researched; secondly in the second comparison all the disturbances of background were removed. The paired samples had same genome, same growth environment and same development stage, so the positional difference was the only difference between them. Therefore, the DEGs identified here could provide breeders reliable candidate genes for plants' drought resistance improvement.

The transcriptome is the set of all messenger RNA molecules in a given organism at a given growth stage, and it is the most important part of genetic regulation in all living things. The clustering result of the transcriptomic data was completely consistent with the phenotype of RDR (Figure 2, Table S1), which also could be found from the experiment of RNA microarrays assay (Table S2, Figure S1). It implied the difference of transcriptomes could represent the difference of varieties with diverse RDR phenotypes, so the transcriptomic analysis was suitable for deep rooting research. Furthermore, all the paired samples from the same variety were always clustered together in the minimum group, suggesting the differences derived from position-specificity were much less than the differences derived from genotype diversity.

The transcriptomic differences among different varieties were diverse. H vs. L contained 13,242 DEGs > H vs. M contained 7,252 DEGs> M vs. L contained 5,248 DEGs (Figure 3B). Therefore, compared with shallow and median rooting varieties, the deep rooting varieties H had the largest transcriptomic specificity. Furthermore, there were 5,192 DEGs could be both found in H vs. L and H vs. M, that was much more than the DEGs (1,507) could be exclusively found in M vs. L. It suggested the similarity between M and L is the largest one among three pair-wise comparisons. As a result, we could speculate the similarity between shallow rooting and median rooting varieties was quite high. From the Figure 2, we could also find most of the median varieties cluster with shallow rooting varieties. In a word, the deep rooting varieties were a special group compared with other rice varieties, and they might have experienced strict selection and developed a set of particular patterns of phenotype, genotype and transcriptome.

The DEGs of deep rooting varieties and shallow rooting varieties were enriched mainly in the pathway of genetic information processing and metabolism (Table 2). It suggested the divergence of deep rooting and shallow rooting rice varieties may be caused by their difference in the genetic information processing and metabolism. However, the DEGs from the paired comparison of shallow roots vs. deep roots were significantly enriched in the metabolism pathways especially energy metabolism (Table S5). It implied the importance of the metabolism process to the deep rooting. Meanwhile, the DEGs of D vs. S also enriched in the genes' collection controlling roots architecture with *P*-value 8.04E-22 (Table S6). It indicated the reliability of the DEGs identified in this study. Furthermore, we found 52 DEGs between deep and shallow roots from the same variety located on the intervals of QTLs controlling RDR that had been identified by our previous work (Lou et al., 2015). We also compared the 1,052 DEGs with the QTLs for root traits contributing to drought resistance like deep root rate, root volume, root length, and root weight in rice (Qu et al., 2008; Courtois et al., 2009; Obara et al., 2010; Uga et al., 2011, 2013b, 2015; Liang et al., 2013; Sthanumoorthy and Tamba, 2013; Wang et al., 2013), and we found 790 DEGs were located on the intervals of the 305 known QTLs reported to be related to the roots architecture and drought resistance. Therefore, the DEGs found here were quite reasonable and can be used for theoretical study and practical utility by roots architecture modification.

WGCNA is a useful way to analyze transcriptomic data to find the network of associated genes with certain functions and even identify new genes. In this study, through WGCNA using all the DEGs of D vs. S, a big module consisted of the top 300 genes' co-expressing network was found (Figure 5). Some hub genes had close correlations with many other genes in this big module. Through annotation, we surprisingly found half of them belong to mitochondria genes and involve in the energy metabolism, like the synthesis of ATP (Table 6). And all the hub genes expressed higher in deep roots than in shallow roots. Meanwhile, through QTTs mapping, two genes involved in the energy metabolism (LOC_Os01g42430 and LOC_Os07g23380) were also identified to be associated with the RDR (Table 5, Table S9). Almost all these hub genes have significantly higher expression in deep roots than that in shallow roots from the same variety. Through KEGG analysis of all the DEGs between deep roots and shallow roots from the same variety, the DEGs were significantly enriched in five energy metabolism pathways (Table S5). Besides the evidence of molecular data, we also found physiological evidence that the deep roots could produce more ATP than shallow roots. Therefore, we can infer the development of the roots may closely relate to energy metabolism. Deeper roots need more energy than shallow roots, or more energy could make roots grow deeper. The amount of energy could decide the distribution of roots. The further studies of these energy metabolism-related candidate genes and the map-based cloning of more RDR-related QTLs would provide more information to elucidate the relationship between energy metabolism and deep root growth.

## Deposited data

The RNA-seq datasets generated in this study have been submitted to NCBI Sequence Read Archive (SRA) with the Bioproject number PRJNA306542 from SRX1547421 to SRX1547494.

## Author contributions

QL and LC performed the experiment, analyzed the data, and drafted the article. HM reviewed and edited the manuscript. KX, TL, and XP contributed to data acquisition. HW, FF, and CS participated in the data analysis and interpretation. LL and YZ conceived and designed the study.

### Conflict of interest statement

The authors declare that the research was conducted in the absence of any commercial or financial relationships that could be construed as a potential conflict of interest.
