# Supplementary material for: Root Transcriptomic Analysis Revealing the Importance of Energy Metabolism to the Development of Deep Roots in Rice (Oryza sativa L.)
Source: Front Plant Sci. 2017 Jul 26;8:1314. doi: 10.3389/fpls.2017.01314 (PMC5526896; doi:10.3389/fpls.2017.01314)
Supplement: Supplementary file 1 [file Presentation1.PDF]

Figure S1 The relationship among the 12 samples. a. Clustering 12 samples using their microarray data; b. The PCA plot of these 12 samples using their microarray data. H is the group of deep rooting rice varieties and L is the group of shallow rooting rice varieties. S are the samples of shallow roots, and D are the samples of deep roots marked by red.

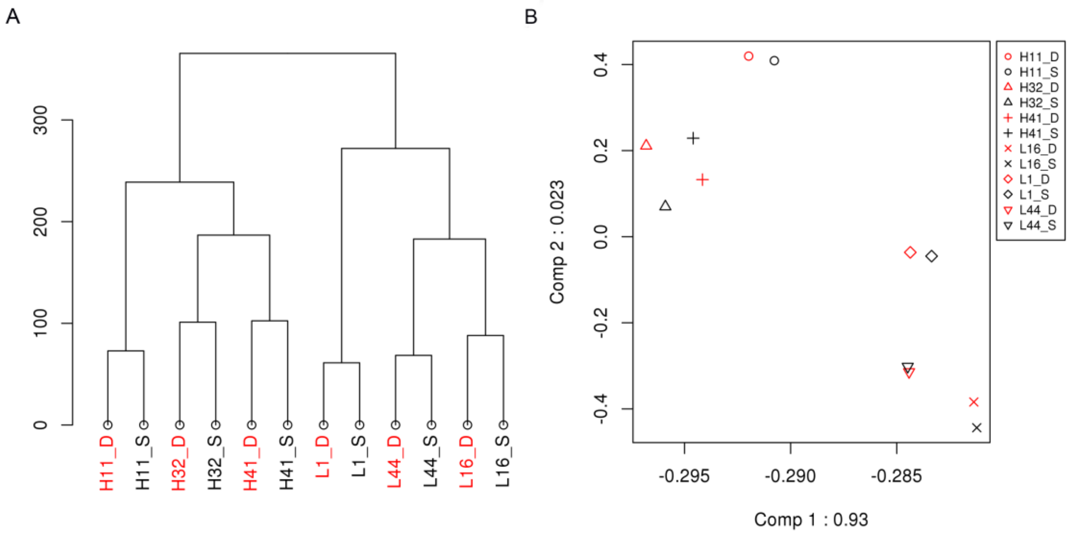

Table S1 The list of samples used for RNA-seq experiment.

No	Name	Subspecies	RDR
H30	Maogu	*Indica*	46.50%
H18	Hongxinnuogu	*Japonica*	46.90%
H38	Miyang93	*Japonica*	47.40%
H27	Hangu	*Indica*	49.80%
H44	BICOPRETO	*Japonica*	50.10%
H34	Hongmigeng	*Japonica*	50.30%
H32	Haoguo	*Indica*	52.00%
H12	CICA4	*Japonica*	52.00%
H5	Handao8	*Japonica*	53.00%
H49	Shanhangu	*Indica*	55.70%
H6	Feidongtangdao	*Japonica*	56.00%
H47	Mowanggu	*Japonica*	58.20%
H43	IPEACO162	*Japonica*	59.80%
H13	Huhan7	*Indica*	59.90%
L47	Hanzhangu	*Indica*	5.20%
L1	Nanxiongzaoyou	*Indica*	6.60%
L44	IR64	*Indica*	6.90%
L36	Daliangdao	*Japonica*	12.10%
L12	Zhenxian232	*Indica*	13.10%
L31	H1K0265	*Indica*	14.40%
L5	Tainong67	*Japonica*	14.50%
L14	Menjiading2	*Indica*	14.70%
L23	Gang46B	*Indica*	15.10%
L26	Naqihangu	*Indica*	15.10%
L42	Haoannong	*Indica*	15.60%
L40	BLCO.BRANCO	*Indica*	15.90%
L16	Gui630	*Indica*	15.90%
L4	Jinxibai	*Indica*	16.30%
L46	CR203	*Indica*	16.80%
M2	Qingsizhan	*Indica*	19.00%
M3	Hanhui3	*Indica*	17.00%
M4	Huhan1B	*Indica*	30.00%
M5	Lvhan1	*Indica*	21.00%
M6	Zhenshan97B	*Indica*	20.00%
M7	Huhan15	*Indica*	25.00%
M8	Ninghui21	*Japonica*	22.00%
M9	Yunlu8	*Japonica*	24.00%

H is the group of deep rooting rice varieties, L is the group of shallow rooting rice varieties and M is the group of median rooting rice varieties.RDR is the ratio of deep roots

Table S2 The list of samples used for microarray test.

**No**	**Name**	**Subspecies**	**RDR**
L1	Nanxiongzaoyou	*indica*	6.6%
L16	Gui630	*indica*	15.9%
L44	IR64	*indica*	6.9%
H11	IAC1	*japonica*	32.5%
H32	Haoguo	*japonica*	52.0%
H41	IRAT109	*japonica*	54.0%

Table S3 The transcriptomic data of the 74 samples.

	Raw Reads	Clean Reads	Mapped reads	Mapping rate	Q20	Q30
Means	4.61E+07	4.31E+07	3.99E+07	92.60%	93.30%	86.87%
Range	2.27E+07	2.16E+07	2.00E+07	7.12%	3.39%	4.90%
Min	3.37E+07	3.11E+07	2.84E+07	88.68%	91.15%	84.02%
Max	5.64E+07	5.27E+07	4.85E+07	95.80%	94.54%	88.92%
Sum	3.41E+09	3.19E+09	2.95E+09			

Table S4 The number of genes differentially expressed in deep and shallow roots sampled from the same variety (FDR<0.05).

Samples group	Number	Up[a]<br>（S/D>1）	Down[b]<br>（S/D<1）
H（14pairs）	599	272	248
L（15pairs）	488	184	244
M（8pairs）	299	197	85

H is the group of deep rooting rice varieties, L is the group of shallow rooting rice varieties and M is the group of median rooting rice varieties. a: was the number of genes expressed higher in shallow roots than in deep roots (equal to S/D>1); b: was the number of genes expressed lower in shallow roots than in deep roots (equal to S/D<1). S and D were the genes’ expression in shallow roots and deep roots, respectively.

Table S5 The KEGG pathway enrichment analysis of 1052 genes differentially expressed between deep and shallow roots from the same variety.

KEGG pathway	P-Value	typeII	typeI
Diterpenoid biosynthesis	7.90E-13	Metabolism of terpenoids and polyketides	Metabolism
Photosynthesis - antenna proteins	2.35E-08	**Energy metabolism**	Metabolism
Phenylpropanoid biosynthesis	1.48E-07	Biosynthesis of other secondary metabolites	Metabolism
Linoleic acid metabolism	0.000755	Lipid metabolism	Metabolism
Cysteine and methionine metabolism	0.001293	Amino acid metabolism	Metabolism
Nitrogen metabolism	0.002091	**Energy metabolism**	Metabolism
Ribosome	0.002241	Translation	Genetic Information Processing
Oxidative phosphorylation	0.004526	**Energy metabolism**	Metabolism
Isoquinoline alkaloid biosynthesis	0.013077	Biosynthesis of other secondary metabolites	Metabolism
Sulfur metabolism	0.016384	**Energy metabolism**	Metabolism
Tyrosine metabolism	0.017732	Amino acid metabolism	Metabolism
Metabolism of xenobiotics by cytochrome P450	0.019257	Xenobiotics biodegradation and metabolism	Metabolism
Drug metabolism - cytochrome P450	0.019257	Xenobiotics biodegradation and metabolism	Metabolism
Pentose and glucuronate interconversions	0.039805	Carbohydrate metabolism	Metabolism
Cyanoamino acid metabolism	0.039805	Metabolism of other amino acids	Metabolism
Arginine and proline metabolism	0.047126	Amino acid metabolism	Metabolism
Photosynthesis	0.047942	**Energy metabolism**	Metabolism
Thiamine metabolism	0.048249	Metabolism of cofactors and vitamins	Metabolism

TypeI was the primary class of KEGG pathway hierarchy, TypeII was the secondary class of KEGG pathway hierarchy. The pathways involved in energy metabolism were marked by boldface.

Table S6 The overlap of the DEGs and the known genes characterized to be related to root architecture.

	Related to root architecture	Not Related to root architecture	Total
DEGs	75	977	1052
Not DEGs	1305	53606	54911
Total	1380	54583	55963

Table S7 The list of 75 DEGs known to control the root architecture.

No	Chr	Start	End	Gene_id	Gene_anno
1	3	8963965	8966806	LOC_Os03g16260	protein kinase putative expressed
2	10	20607467	20608460	LOC_Os10g38660	glutathione S-transferase putative expressed
3	8	23502943	23505310	LOC_Os08g37210	patatin putative expressed
4	3	29110322	29111122	LOC_Os03g50960	LTPL118 - Protease inhibitor%2Fseed storage%2FLTP family protein precursor expressed
5	3	1862352	1864245	LOC_Os03g04070	no apical meristem protein putative expressed
6	4	20534071	20534774	LOC_Os04g33900	ctr copper transporter family protein putative expressed
7	1	29188850	29190965	LOC_Os01g50820	transporter major facilitator family putative expressed
8	2	11997094	12002633	LOC_Os02g20360	tyrosine aminotransferase putative expressed
9	11	21675027	21684774	LOC_Os11g36719	lipoxygenase putative expressed
10	6	21708951	21714106	LOC_Os06g36850	cysteine synthase putative expressed
11	7	24055001	24055950	LOC_Os07g40080	ZOS7-09 - C2H2 zinc finger protein expressed
12	8	17558062	17560192	LOC_Os08g28710	receptor protein kinase CRINKLY4 precursor putative expressed
13	8	27875142	27876599	LOC_Os08g44270	vignain precursor putative expressed
14	10	20862952	20873797	LOC_Os10g39130	OsMADS56 - MADS-box family gene with MIKCc type-box expressed
15	10	20437920	20439182	LOC_Os10g38160	glutathione S-transferase putative expressed
16	11	18382019	18390451	LOC_Os11g31470	expressed protein
17	8	15008763	15009754	LOC_Os08g24790	AIR12 putative expressed
18	4	26099273	26101782	LOC_Os04g44060	aquaporin protein putative expressed
19	2	24683633	24685205	LOC_Os02g40710	ammonium transporter protein putative expressed
20	11	9410401	9411512	LOC_Os11g16970	carnitine racemase like protein putative expressed
21	2	627342	630655	LOC_Os02g02120	OsWAK11 - OsWAK receptor-like protein kinase expressed
22	2	6069031	6075807	LOC_Os02g11760	pleiotropic drug resistance protein putative expressed
23	1	33659782	33672538	LOC_Os01g58240	OsSub6 - Putative Subtilisin homologue expressed
24	2	655324	657245	LOC_Os02g02170	transporter major facilitator family putative expressed
25	2	30206964	30208306	LOC_Os02g49440	dof zinc finger domain containing

					protein putative expressed
26	3	28049441	28053725	LOC_Os03g49260	lipoxygenase putative expressed
27	6	29156425	29157883	LOC_Os06g48200	glycosyl hydrolases family 16 putative expressed
28	1	33691773	33696746	LOC_Os01g58290	OsSub9 - Putative Subtilisin homologue expressed
29	2	19309594	19313057	LOC_Os02g32590	HSF-type DNA-binding domain containing protein expressed
30	1	5236623	5244520	LOC_Os01g10040	cytochrome P450 putative expressed
31	7	17640723	17643252	LOC_Os07g29960	cytochrome P450 putative expressed
32	4	29308245	29314112	LOC_Os04g49150	OsMADS17 - MADS-box family gene with MIKCc type-box expressed
33	11	28215210	28223264	LOC_Os11g46950	wall-associated receptor kinase-like 18 precursor putative expressed
34	3	4913122	4914145	LOC_Os03g09880	AIR12 putative expressed
35	5	26344414	26346889	LOC_Os05g45410	HSF-type DNA-binding domain containing protein expressed
36	10	20633312	20634954	LOC_Os10g38740	glutathione S-transferase putative expressed
37	1	2202264	2203860	LOC_Os01g04800	B3 DNA binding domain containing protein expressed
38	8	23545530	23548154	LOC_Os08g37250	patatin putative expressed
39	10	4075644	4081621	LOC_Os10g07556	wall-associated receptor kinase-like 22 precursor putative expressed
40	1	24075065	24082181	LOC_Os01g42380	pleiotropic drug resistance protein putative expressed
41	4	7250358	7251846	LOC_Os04g13140	vignain precursor putative expressed
42	3	4519405	4525778	LOC_Os03g08754	OsMADS47 - MADS-box family gene with MIKCc type-box expressed
43	5	783045	784097	LOC_Os05g02390	ZOS5-02 - C2H2 zinc finger protein expressed
44	2	10363979	10365536	LOC_Os02g17900	glycosyl hydrolases family 16 putative expressed
45	4	1702003	1710519	LOC_Os04g03796	OsSub37 - Putative Subtilisin homologue expressed
46	1	37146917	37149200	LOC_Os01g63980	ZOS1-17 - C2H2 zinc finger protein expressed
47	8	26332323	26335559	LOC_Os08g41720	auxin efflux carrier component putative expressed
48	5	28047335	28048343	LOC_Os05g48890	fasciclin domain containing protein expressed
49	11	10352224	10376611	LOC_Os11g18366	cycloartenol synthase putative expressed
50	2	29238064	29241762	LOC_Os02g47810	dof zinc finger domain containing

					protein putative expressed
51	9	17903164	17905338	LOC_Os09g29460	homeobox associated leucine zipper putative expressed
52	8	679358	681739	LOC_Os08g02070	OsMADS26 - MADS-box family gene with MIKCc type-box expressed
53	10	3991788	3993648	LOC_Os10g07510	DUF260 domain containing protein putative expressed
54	10	21693776	21694502	LOC_Os10g40520	LTPL145 - Protease inhibitor%2Fseed storage%2FLTP family protein precursor expressed
55	3	34427699	34428613	LOC_Os03g60570	ZOS3-22 - C2H2 zinc finger protein expressed
56	4	27743339	27743734	LOC_Os04g46820	LTPL121 - Protease inhibitor%2Fseed storage%2FLTP family protein precursor putative expressed
57	1	41844009	41845691	LOC_Os01g72140	glutathione S-transferase putative expressed
58	4	4457400	4461845	LOC_Os04g08350	cysteine synthase chloroplast%2Fchromoplast precursor putative expressed
59	1	42080116	42080556	LOC_Os01g72550	OsCML19 - Calmodulin-related calcium sensor protein expressed
60	3	30315455	30318972	LOC_Os03g52860	lipoxygenase putative expressed
61	2	667194	669053	LOC_Os02g02190	transporter major facilitator family putative expressed
62	4	30482082	30483492	LOC_Os04g51460	glycosyl hydrolases family 16 putative expressed
63	6	2949764	2957842	LOC_Os06g06350	AMP-binding enzyme putative expressed
64	2	9764691	9770197	LOC_Os02g17080	OsSub16 - Putative Subtilisin homologue expressed
65	5	605760	606871	LOC_Os05g02070	expressed protein
66	2	28487549	28498680	LOC_Os02g46680	multidrug resistance protein putative expressed
67	4	27740050	27740811	LOC_Os04g46810	LTPL120 - Protease inhibitor%2Fseed storage%2FLTP family protein precursor expressed
68	10	21655392	21656252	LOC_Os10g40420	LTPL138 - Protease inhibitor%2Fseed storage%2FLTP family protein precursor expressed
69	11	28174981	28179995	LOC_Os11g46900	wall-associated receptor kinase 3 precursor putative expressed
70	11	27399319	27406608	LOC_Os11g45280	protein kinase family protein putative expressed

71	2	9712538	9715959	LOC_Os02g17000	OsSub14 - Putative Subtilisin homologue expressed
72	3	34424180	34425104	LOC_Os03g60560	ZOS3-21 - C2H2 zinc finger protein expressed
73	1	43194020	43194896	LOC_Os01g74590	MYB family transcription factor putative expressed
74	7	23559520	23560868	LOC_Os07g39320	homeobox domain containing protein expressed
75	11	28119286	28122173	LOC_Os11g46860	wall-associated receptor kinase-like 4 precursor putative expressed

Table S8 The correlation coefficient of the 49 DEGs' expression in the same variety among three RNA surveying methods.

		RNA-seq		qRT-PCR	
		H32s/d	L16s/d	H32s/d	L16s/d
qRT-PCR	H32s/d	0.80			
	L16s/d		0.75		
Microarray	H32s/d	0.46		0.63	
	L16s/d		0.40		0.43

Table S9 The annotation of the genes identified by QTT mapping.

Gene	Chr	Start	End	Strand	Annotation
LOC_Os01g02280	1	707340	714322	+	Leucine Rich Repeat family protein.
LOC_Os01g15860	1	8931593	8936683	+	pre-mRNA-processing factor 6.
LOC_Os01g42140	1	23886843	23888222	-	expressed protein.
**LOC_Os01g42430**	1	24126549	24129627	+	vacuolar ATP synthase.
**LOC_Os01g65150**	1	37814673	37816775	+	proton-dependent oligopeptide transport.
LOC_Os03g08230	3	4194283	4196467	-	sodium/calcium exchanger protein.
LOC_Os03g44840	3	25297660	25301880	-	choline transporter-related.
**LOC_Os05g49980**	5	28663259	28666983	+	OsFBL26 - F-box domain and LRR containing protein.
LOC_Os08g06810	8	3800784	3803051	+	expressed protein.
LOC_Os09g10860	9	5943545	5943847	-	expressed protein.
**LOC_Os10g28400**	10	14773749	14775841	-	MTN26L4 - MtN26 family protein .

The genes differentially expressed between deep rooting and shallow rooting varieties were highlighted by boldface.

Table S10. The summary of WGCNA in six different groups using all the DEGs expressed differentially between shallow roots and deep roots ($p<0.05$).

Samples group	Number of used DEGs	Number of coexpressed DEGs	Average weight of the top 300 pairs of genes' correlation
Whole	9820	73	0.582
D	9820	77	0.576
S	9820	61	0.438
H	9820	49	0.504
L	9820	68	0.648
M	9820	58	0.618

All the 9820 DEGs between shallow roots and deep roots with $p<0.05$ were used in WGCNA among six different samples groups**.** After analysis, the top 300 pairs of co-expressed genes interactions were chosen for further analysis. Whole is all the 74 samples; S is the shallow roots sample; D is the deep roots sample; H is the group of deep rooting rice varieties; L is the group of shallow rooting rice varieties; and M is the group of median rooting rice varieties.
